# *Phyllanthus emblica* L. Enhances Human Umbilical Vein Endothelial Wound Healing and Sprouting

**DOI:** 10.1155/2013/720728

**Published:** 2013-03-31

**Authors:** Linda Chularojmontri, Maneewan Suwatronnakorn, Suvara K. Wattanapitayakul

**Affiliations:** ^1^Department of Preclinical Sciences, Faculty of Medicine, Thammasat University, Pathum Thani 12121, Thailand; ^2^Department of Pharmacology, Faculty of Medicine, Srinakharinwirot University, Bangkok 10110, Thailand

## Abstract

Endothelial dysfunction is the hallmark of impaired wound healing and increased risk of cardiovascular disease. Antioxidants from natural sources decrease oxidative stress and protect against cellular damage caused by reactive oxygen species (ROS). In this study, we examined the antioxidant constituents and capacity of *Phyllanthus emblica* L. (PE) fruit in freeze-dried power form. The pharmacological properties of PE were investigated using human umbilical vein endothelial cells (HUVECs) in the aspects of endothelial cell proliferation, nitric oxide (NO) production, wound healing, cell migration, *in vitro* angiogenesis, and VEGF gene expression. The ASC content of PE was 1.574% + 0.046% (w/w) as determined by HPLC and the total phenolic content was 36.1% ± 0.7% gallic acid equivalent when measured by Folin-Ciocalteu assay. The FRAP assay revealed a relatively high antioxidant capacity at 3,643 + 192.5 *µ*mole/mg. PE at 0.1 to 10 *µ*g/mL did not significantly influence endothelial cell proliferation, but at higher concentrations PE decreased cell survival to 62%. PE significantly promoted NO production, endothelial wound closure, endothelial sprouting, and VEGF mRNA expression. Therefore, PE is a candidate for antioxidant supplement that promotes endothelial function and restores wound healing competency.

## 1. Introduction

 Endothelial dysfunction is the foremost step in the development of atherosclerosis and causes impairment in cell proliferation, migration, wound healing, and angiogenic function [1]. Pathologic oxidative stress conditions, such as diabetes and cardiovascular disease, potentiate endothelial dysfunction eventually leading to ischemic vascular damage and become impediment to wound healing. Current therapeutic strategies focusing on protection of endothelial oxidative damage, accelerating endothelial wound healing, or promoting angiogenesis may have a role in diabetic and oxidative related vascular disease [2]. 

 Impaired endothelial function is mainly characterized by reduced nitric oxide (NO) production, decreased cell migration to repair the endothelial damage, and being deficient in the capacity to form new collateral vessels. Substances that enhance NO bioavailability and promote vascular endothelial growth factor (VEGF) synthesis are shown to improve wound healing and/or angiogenesis [3, 4]. Several oxidative stress markers such as homocysteine and long-standing hypoxia are known to impair endothelial wound healing while using antioxidants have been proven to improve endothelial function and wound repair [5, 6]. Of these, natural antioxidants are the current main focus in complementary and alternative medicine research. In traditional medicine, *Phyllanthus emblica* L. (PE), an indigenous plant grown in Thailand and some parts of Asia, has long been used as a wound healing agent either in single formulation or combined components in traditional preparations. The effect of PE on wound repair has been demonstrated in rats and the mechanisms involve modulation of collagen synthesis, extracellular matrix (ECM) protein synthesis, and antioxidant status [7, 8]. However, the pharmacological action of PE exclusively on endothelial function, wound repair, and tube formation has not been reported. In this study, we investigated the properties of PE fruits in the aspects of antioxidant capacity, ascorbic acid content, total phenolics level, and vasculogenic property that promoted endothelial NO production, wound healing, and *in vitro* angiogenesis using human umbilical cord vein endothelial cells (HUVECs). 

## 2. Materials and Methods

### 2.1. Chemicals and *Phyllanthus emblica* L. Fruit Extract (PE)

All chemicals were purchased from Sigma-Aldrich (St. Louis, MO, USA) or otherwise indicated. PE fruits were obtained from Nakhon Ratchasima province, Northeast of Thailand. PE fruit juice was extracted from 303 g (57 fresh fruits) using a fruit juice extractor and yielded approximately 2.54 mL/fruit. PE juice was then filtered through 0.2 *μ*m membrane filter and underwent freeze-drying process giving 16.1% yield (w/v). The yellowish dry power of PE was kept at 4°C until use. The aqueous stock solutions at 10 mg/mL were prepared freshly at the time of use in each experiment. 

### 2.2. Ascorbic Acid Content, Antioxidant Activity, and Total Phenolic Compounds of PE

 It is well recognized that PE exerts various biological activities partly due to its antioxidant activity such as ascorbic acid (ASC), polyphenolic compounds, and flavonoids. This study evaluated ASC content which is the major antioxidant component of the extract using HPLC method (Thermo Scientific). Standard curve of ASC was established by measuring the areas under the peak after injecting a series of ASC stock solutions into reversed phase HPLC column (Luna C18, 5 *μ*m, dimension 150 × 4.60 mm, Phenomenex, Thailand) with mobile phase (100 mM phosphate buffer 95% : methanol 5%) at the flow rate of 0.4 mL/min, isocratic elution, and detected by UV absorption at 243 nm [9].

Total antioxidant capacity of PE was determined by Ferric reducing antioxidant power (FRAP) assay. The FRAP assay determined the ability of PE to deliver one electron to Fe^III^-TPTZ (2,4,6-Tri(2-pyridyl)-s-triazine) complex to form a color ferrous ion. Briefly, ten microliters of standard FeSO_4_ or sample solutions were added to a 96-well microplate followed by adding 200 *μ*L of FRAP reagent (acetate buffer 300 mM (pH 3.6), TPTZ 10 mM in HCl 40 mM and FeCl_3_·6H_2_O 20 mM) and the development of blue color was monitored at 595 nm (Synergy, BioTek, USA) [10]. The stability of antioxidant capacity was determined from the dry power of PE samples stored at 4°C for 12 months.

Total phenolic content was evaluated by Folin-Ciocalteu assay using gallic acid (GA) as an assay standard [11]. Briefly, a series of standard GA concentrations (0, 31.25, 62.5, 125, 250, 500, and 1000 *μ*M) or samples at the volume of 1.6 mL were added to a reaction test tubes followed by 100 *μ*L of Folin-Ciocalteu reagent and 300 *μ*L of Na_2_CO_3_ solution. The mixture was incubated at 40°C for 30 min and then cooled down at room temperature for 5 min. The products of phenolic compounds reaction were measured at 756 nm (Shimadzu UV-1601, Japan). The amount of total phenolic compounds was represented as GA equivalence (GAE).

### 2.3. Human Umbilical Vein Endothelial Cell (HUVEC) Culture

Human umbilical cords were collected from the labor room of the university hospital and HUVECs were isolated within 48 h as described previously [12]. Cells were cultured in M199 medium, supplemented with 20% fetal bovine serum (FBS) with antibiotic and antimycotic agents (Invitrogen, USA), in a humidified atmosphere of 95% air and 5% CO_2_ at 37°C. Cells between passages 3 and 5 were used in the experiments and were cultured in low serum medium (1% FBS) during PE incubation or other treatments. 

### 2.4. Cytotoxicity of PE and Ascorbic Acid (ASC)

There have been reported that PE induced apoptosis in many cell types including at least 7 cancer cell lines and primary osteoclasts [13–15]. Therefore, this experiment was aimed to determine nontoxic doses of PE and its major antioxidant ASC for further experiments. HUVECs were treated with various concentrations of PE or ASC for 48 h. Cell survival was evaluated using MTT (3-(4,5-dimethylthiazol-2-yl)-2,5-diphenyltetrazolium bromide) assay and sulforhodamine B (SRB) cytotoxicity assay. For MTT assay, ten microliters of MTT stock solution (5 mg/mL) were added to the culture medium and incubated for 4 h or until dark blue-purple crystalline precipitates were visualized under an inverted microscope. Then, one hundred microliters of DMSO were added to dissolve the formazan products. The plate was then shacked at 200 rpm for 20 min and the relative cell viability was detected absorbance at 550 nm by Synergy plate reader (BioTek, USA). 

SRB assay was performed in 96-well plate after removal of culture medium and washed once with PBS. Two hundred microliters of cold 10% trichloroacetic acid (TCA) were added to the wells to fix cells. Following 30 min incubation at 4°C, TCA was aspirated and the wells were rinsed with water 5 times. Plates were air-dried and 100 *μ*L of SRB solution (0.4% in 1% acetic acid) was added to each well and allowed cells to be stained for 30 min. Cells were then washed with 1% acetic acid until unincorporated dye was removed (approximately 5 times). The plates were air-dried at room temperature for 30 min. The bound SRB dye was solubilized with 200 *μ*L of 10 mM Tris (pH 10.5) for 5 minutes at room temperature. The relative cell viability was determined by absorbance at 510 nm (BioTek, USA).

### 2.5. Endothelial Wound Healing Assay

Scratch wound assay was used to evaluate the ability of PE in promoting endothelial wound *in vitro*. HUVECs were seeded in 6-well plate (Nunc, Thermo Fisher Scientific, USA) at 5 × 10^5^ cells/well in culture medium and allowed the cells to grow to 90% confluence. The culture medium was then replaced with 1% FBS M199 overnight before the scratch wounds were initiated using a sterile 200 *μ*L pipette tip. Photos of wounds were captured by a digital camera (Olympus DP20, Japan) at the same positions at 0, 24, and 48 h. The length of wound confluence was measured by Cell^∧^B program (Olympus, Japan).

### 2.6. Cell Migration Assay

Migration of HUVECs toward a chemoattractant VEGF and testing substances (PE and ASC) in the cell's surrounding environment was determined by Boyden chamber in 24-well plate (Corning, USA). HUVECs were cultured and 200 *μ*L of cells suspension (2 × 10^5^ cells/mL in 1% FBS) was seeded into the chamber with translucent PET membranes 8 *μ*m pore size. Seven hundred and fifty microliters of media containing vehicle (CTRL group), 100 ng/mL VEGF, PE, or ASC were added to the lower chamber. The plate was incubated at 37°C in a CO_2_ incubator for 16 hours. The inserts were then transferred to 0.25% trypsin-EDTA solution and 300 *μ*L of 5 *μ*M calcein-acetoxymethyl ester (CAL-AM, Sigma) was added to the lower chamber and incubated at 37°C in a CO_2_ incubator for 45 minutes. Cells migrated through the micropores used enzyme esterases to hydrolyze the nonfluorescent CAL-AM to highly fluorescent product which was monitored at wavelengths 485/528 nm for excitation/emission, respectively. 

### 2.7. Nitric Oxide (NO) Production

Endothelial NO is important to maintain endothelial health/cell survival and activation cell migration. In this study, the stable product of NO nitrite was evaluated by 2,3-diaminonaphthalene (DAN) assay which is far more sensitive than Griess reaction [16]. This fluorometric method uses the substrate DAN (nonfluorescent) to react with nitrite under acidic conditions to generate 2,3-diaminonaphthotriazole or 1-(H)-naphthotriazole (NATH), the fluorescent product. NATH fluorescent signal is further enhanced by alkalinization of medium to yield 2,3-naphthotriazole anion (NAT). NaNO_2_ standard solution was freshly prepared ranging from 0.13 to 13.33 *μ*M in DMEM before experiment. Seventy-five microliters of standard solutions or the supernatant media from PE or ASC treatments were transferred into 96-well microplate; then, 10 *μ*L of DAN solution (50 *μ*g/mL in 0.62 N HCl) was added to each well for 10 min at room temperature in the dark. Finally, 5 *μ*L of 2.8 N NaOH solution was subsequently added to each well and fluorescence was measured using a fluorescent microplate reader (Synergy HT, BioTek) with excitation/emission wavelengths of 360/460 nm. Nitrite levels of sample were calculated from NaNO_2_ standard curve.

### 2.8. *In Vitro* Angiogenesis Assay

The 3D spheroid-based angiogenesis assay was performed as described by Korff [17]. Each HUVEC spheroid was composed of 500 cells distributed in 1.4% methyl cellulose (Fisher Scientific, USA). Forty-eight spheroids were embedded in 1 mL rat tail collagen (2 mg/mL) and performed in 24-well tissue culture plates. For angiogenic stimulation test, the collagen gels were overlaid with 100 *μ*L medium containing 50 ng/mL vascular endothelial growth factor (VEGF, PeproTech, USA) or PE at various concentrations, and the gels were allowed to be stored at 37°C and 5% CO_2_ for 24 h. Pictures of each spheroid were captured at 0 and 24 h with a digital camera and the cumulative sprout length was measured for at least 10 individual spheroids per treatment (Olympus, Japan). 

### 2.9. VEGF mRNA Expression

 VEGF gene expression was determined based on SYBR-Green fluorescence RT-PCR using commercial kits (BioRad, USA). Total RNA was extracted from cultured HUVECs using Trizol reagent (Invitrogen, USA). Amplification of target genes was initiated by denaturation at 70°C and cooling to 37°C prior to performing reverse transcription at 37°C for 1 h as described in the manufacturer's manual. PCR amplification for *β*-actin and VEGF was carried out in parallel using primer pairs as follows: *β*-actin, forward 5′-GGACTTCGAGCAAGAGATGG-3′, reverse 5′-AGCACTGTGTTGGCGTACAG-3′; VEGF; forward 5′-TATTTTTCTTGCTGCTAAAT-3′, reverse 5′-AATGTTATTGGTGTCTTCAC-3′ (Gen Bank: NM_001025366). Reaction cycles were performed for 40 cycles with two denaturation steps (94°C, 4 min and 94°C, 4 s) followed by annealing at 58°C, 30 s, and extension at 72°C for 10 s. Amplification was terminated with 10 min extension at 72°C (Roter-Gene 6000, Corbett Life Science). Relative mRNA amounts of samples were calculated using the 2^−ΔΔCT^ method. 

## 3. Results

### 3.1. Ascorbic Acid (ASC) Content, Antioxidants Capacity, and Phenolic Content of PE

 PE possessed antioxidant capacity of 3643.3 ± 192.5 *μ*mole/mg which was not significantly changed when kept at 4°C for 12 months (3694.5 ± 105.8 *μ*mole/mg). The amount of total phenolic content which is well corresponding to antioxidant capacity was calculated as 0.361 ± 0.005 mg GAE/mg PE powder. HPLC analysis revealed that PE dry powder consisted of 1.57% ASC (w/w) or equivalent to 2.53 mg/mL fresh juice or approximately 6.42 mg/fruit.

### 3.2. Cytotoxic Effects of PE and ASC

**Figure 1:**
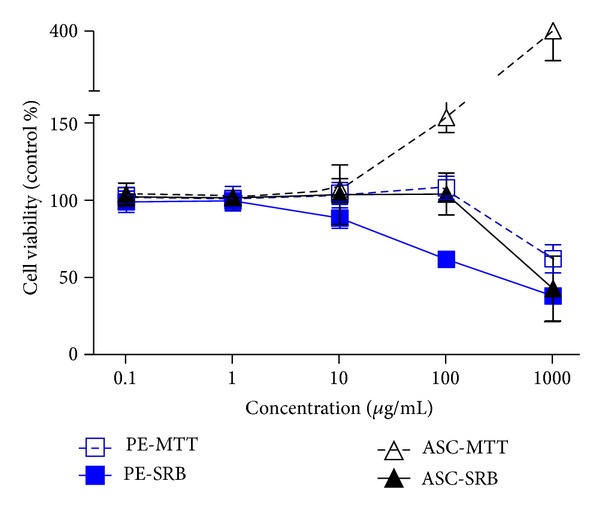
Cytotoxic doses of PE and ASC in HUVECs comparing two methods of cell viability assays. HUVECs were incubated with *Phyllanthus emblica* (PE) or ascorbic acid (ASC) at designated concentrations for 48 h. SRB and MTT assays were performed as described in Section 2.

MTT showed that only PE at 1000 *μ*g/mL caused cytotoxicity while SRB assay detected decreases in cell survival in a concentration-dependent manner beginning from PE at 10 *μ*g/mL (Figure 1). Interestingly, MTT assay showed that ASC at 100 and 1000 *μ*g/mL dramatically increased cell survival by 153.15% and 399.88%, respectively. But SRB assay revealed that ASC 1000 *μ*g/mL decreased cell survival by 42%. Cell survival was then confirmed by cell morphology observed under inverted microscope (data not shown). The cell integrity and morphology of live cells were in accordance with SRB assay; thus noncytotoxic doses of PE and ASC were determined based on SRB assay. 

### 3.3. PE and ASC Enhanced Endothelial Wound Healing

**Figure 2:**
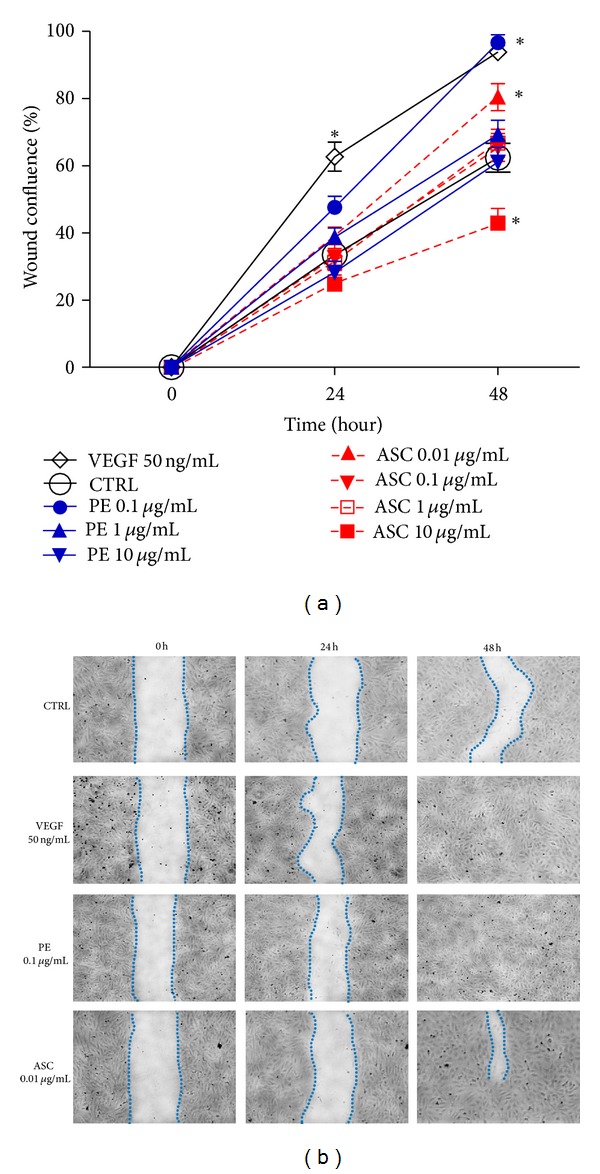
Scratch wound assay. HUVEC wounds were created using pipette tips and the widths of wound areas were measured at the same positions at time 0, 24, and 48 h, after wounds were initiated. (a) PE (0.1–100 *μ*g/mL) or ascorbic acid (ASC, 0.01–10 *μ*g/mL) was incubated with endothelial wounds at time 0. (b) Representative photographs of wound closure at different time points. Dashed line and the wound areas were accentuated for visual purpose. The % wound confluence was calculated compared to vehicle treated group (CTRL). *P < 0.05 versus CTRL.

 Effects of PE and ASC on endothelial wound healing were influenced by the concentrations applied to the scratch wound. At relatively lower concentrations (0.1 *μ*g/mL for PE and 0.01 *μ*g/mL for ASC) these compounds significantly promoted wound confluence only at 48 h while VEGF 50 ng/mL showed significant enhance in wound healing rate since 24 h. PE 0.1 *μ*g/mL completely healed endothelial scratch wound at 48 h which was comparable to wound treated with VEGF. No change was observed in the healing of HUVEC wound when treated with PE at 1, 10 *μ*g/mL and ASC at 0.1, 1 *μ*g/mL (Figure 2). Interestingly, high dose of ASC at 10 *μ*g/mL marked impaired endothelial wound confluence at 48 h.

### 3.4. PE and ASC Promoted Cell Migration

**Figure 3:**
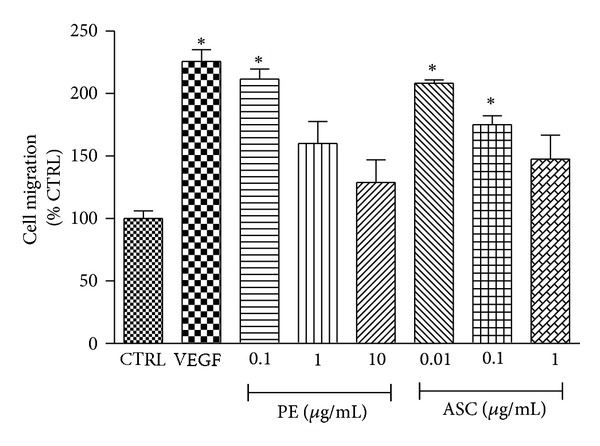
Effect of PE and ASC on cell migration. Comparison of HUVEC migration under influence of the chemotactic factor VEGF (100 ng/mL) and PE (0.1, 1, 10 *μ*g/mL) or ASC (0.01, 0.1, 1 *μ*g/mL) was determined using Boyden chamber assay as described in Section 2. *P < 0.05 versus control.

 Migrated endothelial cells through modified Boyden chambers were measured by reading the fluorescent product of CAL-AM resulted from the metabolism of live cells migrated through the micropores of the upper chamber. Figure 3 demonstrated that PE (0.1, 1, and 10 *μ*g/mL) and ASC (0.01, 0.1, and 1 *μ*g/mL) inversely enhanced cell migration with respect to increasing doses. The lowest two concentrations of PE and ASC used in this experiment significantly promoted endothelial cell migration while the maximum responses were obtained at the degree comparable to the action of the chemoattractant VEGF (100 ng/mL).

### 3.5. Effects of PE and ASC on NO Production

**Figure 4:**
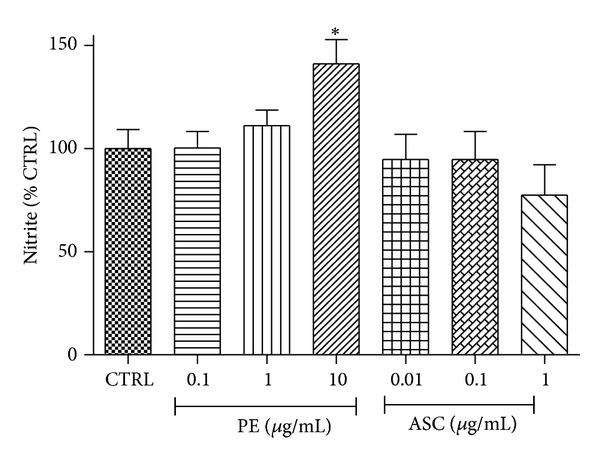
Effects of PE and ASC on nitric oxide levels. The stable product of nitric oxide, nitrite, was determined using DAN assay. Changes in the levels of nitrite in culture media were analyzed at 48 h following PE or ASC treatment as described in Section 2. *P < 0.05 versus CTRL.

 It is well established that NO promotes HUVEC migration. Here we evaluated the stable product of NO nitrite in the culture media of cells treated with PE or ASC corresponding to the doses that shown to promote endothelial migration. It appeared that significant increase in NO production was observed only in HUVEC treated with PE at 10 *μ*g/mL whereas PE at lower doses (0.1 and 1 *μ*g/mL) or all doses of ASC (0.01, 0.1, and 1 *μ*g/mL) did not change nitrite levels (Figure 4). 

### 3.6. Low Doses of PE Promoted Endothelial Sprouting

**Figure 5:**
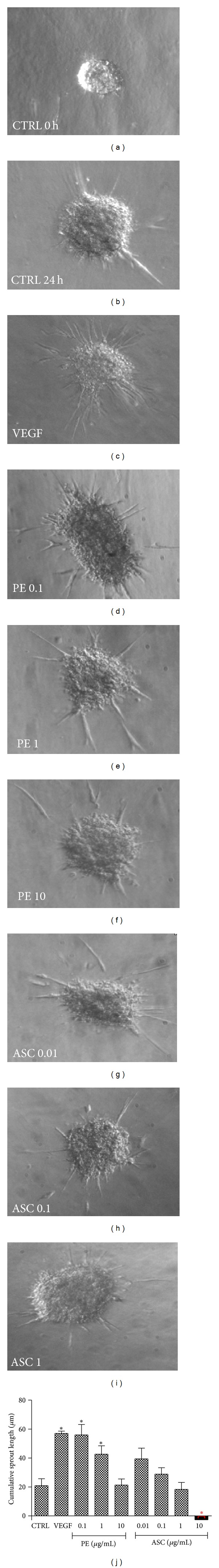
Effects of PE and ASC on endothelial sprouting. Evaluation of *in vitro* model of spheroid angiogenesis was performed 24 h after spheroids were embedded in the collagen matrix containing vehicle (CTRL), VEGF 100 ng/mL (VEGF), PE (0.1, 1, and 10 *μ*g/mL), and ascorbic acid (ASC: 0.01, 0.1, and 1 *μ*g/mL). Cumulative sprout length per spheroid was determined using an image program Cell^∧^B as described in Section 2. (a) to (i) are representative photographs of spheroids with different treatments indicated in the figure; (j) bar graph demonstrates cumulative sprout length of all the treatments. *P < 0.05 versus control.

 PE enhanced endothelial sprouting from spheroids was observed at 0.1 and 1 *μ*g/mL which was similar to its effect on endothelial wound closure. Low PE doses promoted endothelial sprouting but no significant difference from CTRL group was observed at the higher concentration 10 *μ*g/mL (Figure 5). Effects of ASC on endothelial sprout length were inversely related to stepwise increases in concentrations used in the experiment (0.01, 0.1, and 1 *μ*g/mL) while ASC at 10 *μ*g/mL significantly suppressed *in vitro* angiogenesis. 

### 3.7. VEGF mRNA Expression

**Figure 6:**
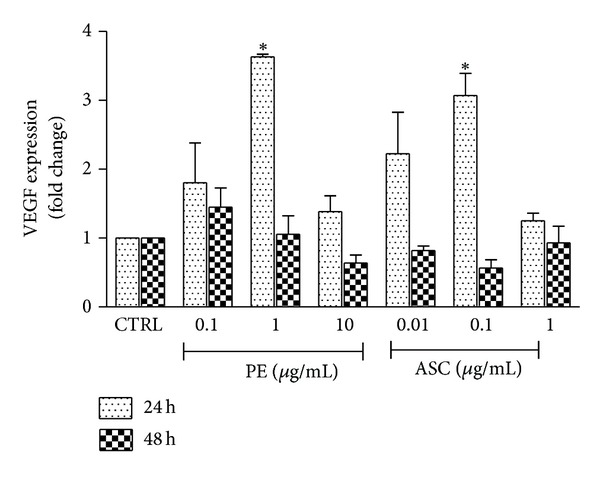
Effects of PE and ASC on VEGF mRNA expression. Changes in VEGF mRNA expression at 24 and 48 h following PE or ASC treatment were determined by real-time PCR as described in Section 2. *P < 0.05 versus CTRL.

 The expression of VEGF mRNA was determined at 24 and 48 h following PE or ASC treatment (as shown in Figure 6). At 24 h after treatment, PE (1 *μ*g/mL) and ASC (0.1 *μ*g/mL) significantly enhanced VEGF mRNA expression by approximately 3.5- and 3-fold, respectively, while other concentrations did not significantly alter VEGF expression. No change was observed in VEGF gene expression when detected at 48 h.

## 4. Discussion 

Antioxidant diets and supplements have increasingly become an important strategy to prevent or slow deterioration of vascular endothelium in well-recognized high oxidative stress conditions such as diabetes and cardiovascular disease. PE is one of the most studied natural antioxidants beneficial to endothelial health in several models of oxidative damage both *in vitro* and *in vivo* [18, 19]. Here we found that PE promoted endothelial wound healing, cell migration, nitric oxide production, and endothelial sprouting which are crucial in endothelial cell function and restoration of endothelial integrity following oxidative damage.

PE is known to contain high antioxidant capacity among medicinal plants and most antioxidant properties found are commonly contributed by ASC and polyphenolic constituents including polyphenols which are best defined in the human diet [20]. The ASC content of PE sample falls into the range of 1.1%–1.7% (w/w) reported by others [21]. Consuming nine PE fruits or 25 mL of fruit juice could receive sufficient ASC at recommended daily intake (RDI) for Thai people (60 mg). This study also revealed that PE fruit juice when kept in dry powder at 4°C retained antioxidant activity for at least 1 year as evaluated by FRAP assay and total phenolic content. This data suggest that preparing PE fruit juice in powder form may be suitable to maintain antioxidant property of the product if further used for food processing and nutrition industry. 

Prior to further study for other pharmacological activities, endothelial cell survival was evaluated after being exposed to varied concentrations of PE. Several methods are available to evaluate cell survival or cell death. The two colorimetric MTT and LDH assays and the two fluorometric assays resazurin and CFDA-AM are among the most assays that used cytotoxicity assays in the field of pharmacology and ecology [22]. Selecting appropriate assay is based on detection mechanism, specificity, and sensitivity. This study first used MTT assay to evaluate the toxicity of PE in HUVEC. It appeared that there might be an interaction between MTT reagent and chemical components in PE. At high concentrations of PE and ASC, the absorbance readings did not correlate with cell viability as evaluated under inverted microscope. As a result, SRB assay was introduced to evaluate cell survival which has been proven to be more sensitive to cell death than MTT assay [23]. PE induced cytotoxicity in endothelial cell culture at concentrations above 10 *μ*g/mL; therefore, PE concentrations used in this study were chosen to be lower than the toxic level. It is critical to choose appropriate cell viability assay for medicinal plant research since this may influence study design and hence interpretations of results from the study, especially when cell death accounts for the inhibitory effect such in wound healing or angiogenesis assay. 

PE accelerated endothelial wound closure only at low concentration (0.1 *μ*g/mL) while higher concentrations did not have any significant effect. PE inclined to cause cytotoxicity at higher concentrations observed in this study and others. For example, the plant extract induced cell death or apoptosis in many human carcinoma cell lines at the range of 50–200 *μ*g/mL [14, 24]. Nonetheless, it also depends on the method of plant extraction and preparation. The mechanism of wound healing is complex but when exclusively considered at endothelial layer there are two main factors involved, that is, cell proliferation and cell migration. Increased cell proliferation may not contribute to the healing effect since no significant change in cell number was observed at this PE concentration. On the other hand, increased cell migration could be the mechanism underlining accelerated wound closure induced by PE where the migration effect correlated well with wound closure phenomenon. Likewise, ascorbic acid at concentrations closely related to its composition ratio in PE demonstrated similar effect on endothelial wound healing and cell migration. However, high dose of ASC (10 *μ*g/mL) inhibited scratch wound closure and angiogenesis which is similar to a report by Mikirova et al. [25] in that high amount of ASC (3 mg/mL) inhibited *in vitro* tube formation and at higher concentrations (10–20 mg/mL) ASC inhibited cell migration 50–60% at 8 h. Thus, it is possible that PE promoted endothelial wound healing and sprouting, at least in part, through the action of ASC at low concentrations. 

 The main characteristic of endothelial dysfunction is the reduction of nitric oxide (NO) bioavailability. Although the dose of PE that increased NO production did not relate to its effect on endothelial wound healing or sprouting, this may have significant impact on maintaining vascular homeostasis and providing microenvironment that can partly restore wound healing deficit. Moreover, nitrate and nitrite have been shown to undergo reduction to NO in the vasculature [26]. It is possible that PE may promote endothelial function at the doses different from other effects and this effect is unlikely caused by ASC constituent but rather induced by other active constituents in PE such as gallic acid [27]. Nonetheless, determinations of changes in eNOS or iNOS expression are needed to confirm the influence of PE on NO production.

VEGF is an important growth factor for angiogenesis in promoting cell proliferations and cell migration through the signaling of VEGF receptors (VEGFR). Upon binding of VEGF to VEGFR, the protein-tyrosine kinase receptor undergoes autophosphorylation and sends signals to PI3K/AKT, resulted in increased NO production (through eNOS) and thereby enhancing cell permeability, vasodilation, and cell migration and survival. The VEGF activation cascade also includes Cd42/p38/MAPKAP-K2 pathway where it activates actin polymerization, stress fiber formation that renders the cell to migrate [28]. Signaling of  *α*_*v*_ *β*_3_  integrin also participates in cell migration through the activation of FAK (phosphorylation at Ser732) that leads to focal adhesion turnover and hence cell migration. Our results did not show correlation between the concentrations of PE induced NO production and the doses that promoted *in vitro* angiogenesis. Thus, it is possible that PE NO alone may not be the sole mechanism that induces cell migration and endothelial sprouting or that the kinetics of NO in the mode of paracrine effect escape sensitivity of assay. Similarly, changes in VEGF mRNA expression did not correlate well in the direction that favors the explanation of NO action. The interpretation of VEGF mRNA expression is complicated due to the intrinsic activity of test substances, experiment conditions, and times of detection. For instance, phorbol-12-myristate-13-acetate (PMA) maximally stimulated VEGF gene expression in HUVEC at 3 h and returned to baseline level within 12 h while hypoxia showed peak VEGF mRNA levels at 48 h [29]. Nonetheless, this study is the first to report the effect of PE on VEGF expression and this is substantiated by its effect on accelerated wound healing effect [7]. Interestingly, the inhibitory effect on VEGF expression is observed when either high dose of ASC was applied to endothelial cells or malignant cells and appeared to correspond to antiangiogenesis effect [30, 31]. This may be due to intracellular redox balance and the crosstalk between oxidative stress signaling and angiogenesis activation cascade [32]. Given that PE possesses high antioxidant capacity, PE might influence cellular redox status which may relate to angiogenesis in the condition that lacks influence from additional growth factors in the culture system.

One advantage using medicinal plant in crude form is the synergistic effect among different chemical constituents. In many cases, purified fractions or single chemicals demonstrate less effective than the crude extract when used at equivalent doses. For example, there are pharmacodynamic synergies among *Cinchona alkaloids* and pharmacokinetics interactions between *Artemisia annua* tea that the crude extracts decrease IC50 by many folds for antimalarial effect when compared to using single compounds alone [33]. The mechanism of synergy is not fully understood but it involves the action at different pharmacological targets in the way that the operations are in parallel. In the case of PE, the concentrations that demonstrated significant changes from each experiment are incongruent in some parts. A certain set of components in PE may be responsible for the activation of NO production while another separate group of compounds work in the same pharmacological targets toward cell migration and endothelial tube formation. Accordingly, further studies to identify active ingredient(s) and the precise mechanism of PE induced endothelial migration and differentiation are warranted.

## 5. Conclusion

Endothelial dysfunction and endothelial damage are important risk factors associated with pathogenesis of impaired wound healing and cardiovascular disease. Consuming antioxidants and compounds that activate endothelial wound healing during oxidative stress may promote endothelial health and favor cardiovascular risk reduction. This study demonstrated that PE possessed high antioxidant capacity and enhanced endothelial wound healing and sprouting at low concentrations, but the opposite effects were observed when investigated at high concentration. These beneficial effects on endothelial cells are partly due to its antioxidant constituent ascorbic acid. Therefore, it is important to consider this dose-related biphasic effects when designing experiments and applying information to clinical studies in order to obtain desirable pharmacological activities.
